# Clinical results of carbon-ion radiotherapy for stage I non-small cell lung cancer with concomitant interstitial lung disease: a Japanese national registry study (J-CROS-LUNG)

**DOI:** 10.1093/jrr/rrad008

**Published:** 2023-04-07

**Authors:** Naoko Okano, Hiroaki Suefuji, Mio Nakajima, Sunao Tokumaru, Nobuteru Kubo, Daisaku Yoshida, Osamu Suzuki, Hitoshi Ishikawa, Miyako Satouchi, Haruhiko Nakayama, Yoshiyuki Shioyama

**Affiliations:** Gunma University Heavy Ion Medical Center, 3-39-15 Showa-machi, Maebashi, Gunma 371-8511, Japan; Ion Beam Therapy Center, SAGA-HIMAT Foundation, 3049, Koga-machi, Tosu, Saga 841-0071, Japan; National Institutes for Quantum and Radiological Science and Technology, 4-9-1, Anagawa, Inage-ku, Chiba, Chiba 263-8555, Japan; Department of Radiology, Hyogo Ion Beam Medical Center, 1-2-1 Kouto, Shingu-cho, Tatsuno, Hyogo 679-5165, Japan; Gunma University Heavy Ion Medical Center, 3-39-15 Showa-machi, Maebashi, Gunma 371-8511, Japan; Kanagawa Cancer Center, 2-3-2 Nakao, Asahi-ku, Yokohama, Kanagawa 241-8515, Japan; Osaka Heavy Ion Therapy Center, 3-1-10 Otemae, Chuo-ku, Osaka, Osaka, 540-0008, Japan; National Institutes for Quantum and Radiological Science and Technology, 4-9-1, Anagawa, Inage-ku, Chiba, Chiba 263-8555, Japan; Department of Thoracic Oncology, Hyogo Cancer Center, 13-70, Kitaoji-cho, Akashi, Hyogo 673-8558, Japan; Department of Thoracic Surgery, Kanagawa Cancer Center, 2-3-2, Nakao, Asahi-ku, Yokohama, Kanagawa 241-8515, Japan; Ion Beam Therapy Center, SAGA-HIMAT Foundation, 3049, Koga-machi, Tosu, Saga 841-0071, Japan

**Keywords:** non-small cell lung cancer (NSCLC), carbon-ion radiotherapy (CIRT), stage I, interstitial pneumonitis, Japan Carbon-ion Radiation Oncology Study Group (J-CROS)

## Abstract

Anti-cancer treatments for lung cancer patients with interstitial lung disease (ILD) are challenging. The treatment options for ILD are often limited because of concerns that treatments can cause acute exacerbation (AE) of ILD. This study aimed to analyze the outcomes of carbon-ion radiotherapy (CIRT) for stage I non-small cell lung cancer (NSCLC) with ILD, using a multi-institutional registry. Patients with ILD who received CIRT for stage I NSCLC in CIRT institutions in Japan were enrolled. The indication for CIRT was determined by an institutional multidisciplinary tumor board, and CIRT was performed in accordance with institutional protocols. Thirty patients were eligible. The median follow-up duration was 30.3 months (range, 2.5–58 months), and the total dose ranged from 50 Gy (relative biological effectiveness [RBE]) to 69.6 Gy (RBE), and five different patterns of fractionation were used. The beam delivery method was passive beam in 19 patients and scanning beam in 11 patients. The 3-year overall survival (OS), cause-specific survival, disease-free survival (DFS) and local control (LC) rates were 48.2%, 62.2%, 41.2% and 88.1%, respectively. Grade > 2 radiation pneumonitis occurred in one patient (3.3%). In conclusion, CIRT is a safe treatment modality for stage I NSCLC with concomitant ILD. CIRT is a safe and feasible treatment option for early lung cancer in ILD patients.

## INTRODUCTION

Anti-cancer treatments for lung cancer patients with interstitial lung disease (ILD) are challenging because modalities such as surgery, chemotherapy, immunotherapy and radiotherapy can cause acute exacerbation (AE) of ILD [1–12]. Stereotactic body radiotherapy (SBRT) using X-rays for stage I non-small cell lung cancer (NSCLC) has been reported to be safe with favorable tumor control [13–15]. However, patients with concomitant ILD have a higher risk of developing both radiation pneumonitis and AE of ILD, which can be fatal [16–20]. Therefore, SBRT is often contraindicated for patients with ILD. Carbon-ion radiotherapy (CIRT) has been used for stage I NSCLC. Compared with X-rays, carbon ions have higher linear energy transfer and larger relative biological effectiveness (RBE) [21, 22]. The physical characteristics of carbon ions, such as its Bragg peak and small lateral scattering, are theoretically superior to those of X-rays, allowing carbon ions to provide a more localized delivery of the radiation dose. With this benefit, CIRT for stage I NSCLC has demonstrated favorable local control (LC) while minimizing damage to normal lung tissues [23–25]. However, there have been few reports on the safety and efficacy of CIRT for NSCLC with concomitant ILD, and these reports have been from single institutions [26, 27]. Thus, this study aimed to evaluate the effects of ILD on the clinical outcomes of CIRT for stage I NSCLC, using a Japanese multicenter prospective registry.

## MATERIALS AND METHODS

### Study design and patients

This was a multicenter, prospective observational registry conducted by The Japan Carbon-ion Radiation Oncology Study Group (J-CROS). The J-CROS is a study group comprising all seven CIRT institutions in Japan. Among these, the National Institutes for Quantum and Radiological Science and Technology (Chiba), Gunma University Heavy Ion Medical Center (Gunma), Ion Beam Therapy Center, SAGA HIMAT Foundation (HIMAT, Saga), Hyogo Ion Beam Medical Center (Hyogo) and Ion-beam Radiation Oncology Center in Kanagawa (Kanagawa) participated in the current multi-institutional study on NSCLC. The other two institutions did not participate because carbon-ion beam was not used for NSCLC due to the shortage of time after installation.

This study involved patients with stage I NSCLC treated with CIRT between May 2016 and June 2018. The inclusion criteria were history of ILD and stage I (T1-2aN0M0) NSCLC based on the TNM Classification of Malignant Tumors, 8th Edition [28]. The Staging of the tumor was determined by chest and abdominal computed tomography (CT); whole-brain magnetic resonance imaging or CT; and ^18^F-fluorodeoxyglucose positron emission tomography. The eligibility criteria for CIRT and lobectomy were decided by a multidisciplinary tumor board in each institution. Complications of ILD were determined either by a respiratory physician’s diagnosis or confirmation by the multidisciplinary tumor board. Toxicities were evaluated using the Common Terminology Criteria for Adverse Events version 5.0 [29].

This study was approved by the institutional review board (Approval No.: UMIN000024709) and was conducted according to the tenets of the Declaration of Helsinki. Written informed consent was obtained from all patients.

### Treatment protocol

CIRT was performed in accordance with institutional protocols. The target volume and dose constraints for organs at risk were determined in accordance with each protocol. Typically, the clinical target volume (CTV) was defined as the gross target volume (GTV) with a 0–5 mm margin. The internal margin was set considering respiratory movement in each direction, as determined from the four-dimensional CT. Finally, the planning target volume (PTV) was created by adding the internal and setup margins to the CTV. Dose prescriptions for CIRT was described as Gy(RBE), defined as the physical dose (Gy) multiplied by the RBE value of the carbon ions [22]. The biological dose was calculated using the biological near infrared spectroscopy model, based on the linear-quadratic model. The dose was administered to the isocenter of the PTV.

### Follow-up

Patients underwent thoracic CT scan and blood analyses every 3 months for a year after CIRT. After 1 year, the frequency was updated to once every 6 months, and the examinations and consultations continued for 5 years after the end of radiotherapy. Additional examinations were performed when recurrence was suspected. The follow-up schedule was adjusted according to the treatment schedule and the patient’s general condition. Treatment toxicity was evaluated according to the Common Terminology Criteria for Adverse Events (CTCAE) version 4.0.

### Statistical analysis

Overall survival (OS) was measured as the time from the date of initial CIRT to the date of death due to any cause or date of the last follow-up, whichever came first. Disease-free survival (DFS) was measured as the time from the date of initial CIRT to the date of death, last follow-up, or tumor relapse metastatic or locally, whichever came first. LC was measured from the date of initial CIRT to the date of the first local progression in the irradiated area or the date of the last follow-up. OS, DFS and LC curves were estimated using the Kaplan–Meier method and were statistically compared between the two groups using the log-rank test. All statistical analyses were performed using SPSS (version 26; SPSS Inc., Chicago, IL, USA). A *P* < 0.05 was considered statistically significant.

## RESULTS

A total of 30 patients were included in this study. The patient characteristics are shown in Table 1. From these patients, five (16.7%) were candidates for lobectomy while the remaining 25 (83.3%) were not candidates for the surgical procedure. The tumor size was ≤3 cm in 24 patients (80%), but 83.3% of the tumors demonstrated solid tumor without ground glass area. There were 17 patients who were not diagnosed pathologically. The median follow-up period was 30.3 months. The treatment method and dose are shown in Table 2. The prescribed dose and fractionation differed among the study institutions.

**Table 1:** Patient characteristics

		(*n* = 30)	
Age (years)			
Median (range)		77(62–86)	
Sex			
Male		28	93.3%
Female		2	6.7%
ECOG performance status score			
0		12	40%
1		15	50%
2		2	6.7%
3		1	3.3%
Smoking history			
Yes		3	10%
No		25	83.3%
Unknown		2	6.7%
Eligibility for lobectomy			
Yes		5	16.7%
No		25	83.3%
T category (UICC 8th)			
T1a		2	6.7%
T1b		7	23.3%
T1c		15	50%
T2a		6	20%
Tumor size (cm)			
Median (range)		2.3(0.8–3.7)	
Consolidation/tumor ratio			
<50%		3	10%
50–99%		2	6.7%
100%		25	83.3%
Histology			
Squamous cell carcinoma		4	13.3%
Adenocarcinoma		5	16.7%
Non-small cell lung cancer		4	13.3%
Clinical diagnosis		17	56.7%
Pre-treatment vital capacity (L)			
Median (range)		2.77(1.31–3.7)	
Pre-treatment FEV1.0 (L)			
Median (range)		1.94(0.95–2.85)	
Follow-up duration (months)			
Median (range)		30.3(2.5–58.0)	

Abbreviations: ECOG = Eastern Cooperative Oncology Group, UICC 8th = Union for International Cancer Control (8th edition).

**Table 2:** Details of CIRT

Prescribed dose [Gy (RBE)]			
50.0 Gy (RBE) in 1 fraction		11	36.7%
60.0 Gy (RBE) in 4 fractions		10	33.3%
64.0 Gy (RBE) in 12 fractions		1	3.3%
66.0 Gy (RBE) in 12 fractions		1	3.3%
69.6 Gy (RBE) in 12 fractions		7	23.3%
Irradiation method			
	Passive	19	63.3%
	Scanning	11	36.7%

Abbreviations: RBE = relative biological effectiveness

**Fig. 1:**
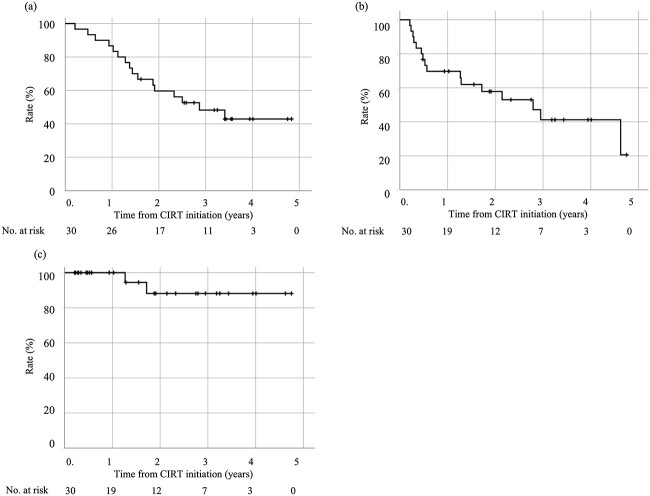
Treatment outcomes of CIRT for stage I NSCLC with concomitant ILD. (a) OS rate. (b) DFS rate. (c) LC rate.

Overall, 16 patients died; among them, seven patients died from lung cancer and nine patients died from other diseases. The OS, DFS and LC values are shown in Fig. 1. The 3-year OS, DFS and LC were 48.2%, 41.2% and 88.1%, respectively. Table 3 shows the results of the analysis of the effect of patient background and treatment factors on OS. No significant influencing factors were identified. With respect to adverse events, no patient developed Grade 4 or 5 adverse events. Only one patient (3.3%) developed Grade 3 pneumonitis, but no other Grade 2 or 3 adverse event occurred.

**Table 3:** Influencing factors of OS

factors	No of patients		3-year OS	*P*-value
COPD	Yes	18	61.1%	0.129
	No	12	19.4%	
DM	Yes	4	25%	0.162
	No	26	51.7%	
Heart disease	Yes	8	50%	0.973
	No	22	47.4%	
Anticoagulant use	Yes	5	40%	0.707
	No	23	50.1%	
Operability	Yes	5	80%	0.124
	No	25	41.9%	
Age (years)	<75	12	37.5%	0.678
	≥75	18	54.5%	
T category	T1	24	57.8%	0.201
	T2	6	33.3%	
Double cancer	Yes	15	44%	0.74
	No	15	53.3%	
Total dose	<64	21	44.9%	0.642
	>64	9	55.6%	
Treatment method	Passive	19	47.4%	0.94
	Scanning	11	46.8%	

Abbreviations: COPD = chronic obstructive pulmonary disease, DM = diabetes mellitus

## DISCUSSION

The safety and efficacy of CIRT for NSCLC patients with concomitant ILD are yet to be clarified. This study found that CIRT was able to safely and effectively treat stage I NSCLC patients with ILD. The 3-year OS, DFS and LC were 48.2%, 41.2% and 88.1%, respectively. Sixteen patients died; among them, seven patients died from lung cancer and nine patients died from other diseases. Lung toxicity occurred in only 1/30 patients. To the best of our knowledge, this study is the first multi-institutional report of CIRT for stage I NSCLC with ILD.

Previous studies have also reported inferior posttreatment survival in patients with ILD than in patients without ILD. Regarding SBRT, the 3-year OS ranges from 0% to 53.8% in ILD patients [19, 30, 31]. A similar trend was demonstrated in a surgical series. Sekihara *et al.* [32] reported that in patients with stage I lung cancer who underwent complete resection, the 5-year OS was significantly higher in the non-ILD group (84.6%) than in the ILD group (44%). In CIRT, OS was worse in the ILD group than in the non-ILD group (3-year OS: 59.7% vs 83.2%) [27]. On the other hand, patients with ILD have poor prognoses due to the ILD itself. Natsuizaka *et al.* [33] reported a median survival for patients with idiopathic pulmonary fibrosis of 35 months. In the current study, the 3-year OS was 48.2% in patients with ILD. As for local effects, the 3-year LC was 88.1% in this study. The local efficacy was comparable to previously reported SBRT and CIRT with LC of 71.4–94%, although there are differences in background tumor factors [19, 27, 30]. Furthermore, patients with ILD reported higher incidence of death due to primary lung cancer than those without ILD [27, 32]. To summarize, the lower OS in this study is related to the poor prognosis of ILD itself and the increased number of primary deaths observed in cases with ILD complications. These results are comparable to other reports.

Studies on CIRT have reported that operability, T-category, and other factors affect the OS [23, 25]. In this study, there were no factors that affected on OS in the patient background or the treatment factors. Possible reasons for the lack of significant differences could be the limited number of patients involved in our study.

NSCLC patients with concomitant ILD are at high risk of severe AEs after SBRT. In retrospective studies on SBRT for NSCLC patients with concomitant ILD, the incidence rates of Grade ≥ 2 and Grade ≥ 3 radiation pneumonitis ranged from 19% to 50% and from 10% to 38.9%, respectively [19, 30, 31]. Meanwhile, the corresponding rates of Grade ≥ 2 and Grade ≥ 3 radiation pneumonitis in CIRT for NSCLC patients with concomitant ILD were 7.7% and 3.8%, respectively [27]. In the current study, the incidence of Grade ≥ 2 and Grade ≥ 3 radiation pneumonitis was 3.3% and 3.3%. These incidence rates were lower than those of SBRT-induced radiation pneumonitis in patients with ILD. Carbon-ion beams realizes conformal dose distribution and normal lung tissue by its physical features. Therefore, the lower incidence rates could be attributed to the better dose distribution in CIRT. The mean lung dose was defined as the average dose to the whole lung. V*n* describes the fraction of the volume irradiated above *n* Gy or *n* Gy (RBE). A previous dosimetric study comparing dose-volume histogram parameters demonstrated that the values of mean lung dose, V5, V20 and V30 were significantly lower in CIRT than in SBRT [34].

The limitations to our study should be mentioned. First, the diagnostic criteria for ILD were not standardized. Second, the number of patients was small. Despite these limitations, to our best knowledge, this was the first multicenter study to report the outcomes of CIRT for stage I NSCLC patients with ILD. The findings provide valuable evidence to guide clinical practice.

In conclusion, CIRT is a safe and feasible treatment option for early lung cancer in ILD patients as it provides good LC without increasing adverse events. However, CIRT for Stage I NSCLC patients with ILD did not improve OS as compared to other modalities. The reasons for the decrease in OS need to be clarified and studied in future prospective studies.

## Data Availability

The datasets in this study belong to JCROS, and permission to use the data must be obtained from the co-authors and JCROS.

## CLINICAL TRIAL REGISTRATION NUMBER

UMIN000024709.
